# 3-Tesla magnetic resonance imaging improves the prostate cancer detection rate in transrectral ultrasound-guided biopsy

**DOI:** 10.3892/etm.2014.2061

**Published:** 2014-11-11

**Authors:** JIE CHEN, XIAO-LEI YI, LI-XIN JIANG, REN WANG, JUN-GONG ZHAO, YUE-HUA LI, BING HU

**Affiliations:** 1 Department of Ultrasound in Medicine, Shanghai Jiaotong University Affiliated Sixth People’s Hospital, Shanghai Institute of Ultrasound in Medicine, Shanghai 200233, P.R. China; 2 Department of Radiology, Shanghai Jiaotong University Affiliated Sixth People’s Hospital, Shanghai 200233, P.R. China

**Keywords:** prostate cancer, transrectal ultrasound, magnetic resonance imaging, biopsy

## Abstract

The detection rate of prostate cancer (PCa) using traditional biopsy guided by transrectal ultrasound (TRUS) is not satisfactory. The aim of this study was to determine the utility of 3-Tesla (3-T) magnetic resonance imaging (MRI) prior to TRUS-guided prostate biopsy and to investigate which subgroup of patients had the most evident improvement in PCa detection rate. A total of 420 patients underwent 3-T MRI examination prior to the first prostate biopsy and the positions of suspicious areas were recorded respectively. TRUS-guided biopsy regimes included systematic 12-core biopsy and targeted biopsy identified by MRI. Patients were divided into subgroups according to their serum prostate-specific antigen (PSA) levels, PSA density (PSAD), prostate volume, TRUS findings and digital rectal examination (DRE) findings. The ability of MRI to improve the cancer detection rate was evaluated. The biopsy positive rate of PCa was 41.2% (173/420), and 41 of the 173 (23.7%) patients were detected only by targeted biopsy in the MRI-suspicious area. Compared with the systematic biopsy, the positive rate was significantly improved by the additional targeted biopsy (P=0.0033). The highest improvement of detection rate was observed in patients with a PSA level of 4–10 ng/ml, PSAD of 0.12–0.20 ng/ml^2^, prostate volume >50 ml, negative TRUS findings and negative DRE findings (P<0.05). Therefore, it is considered that 3-T MRI examination could improve the PCa detection rate on first biopsy, particularly in patients with a PSA level of 4–10 ng/ml, PSAD of 0.12–0.20 ng/ml^2^, prostate volume of >50 ml, negative TRUS findings and negative DRE findings.

## Introduction

Transrectal ultrasound (TRUS)-guided biopsy is the most widely used method for the histological diagnosis of prostate cancer (PCa), which provides real-time imaging of the prostate at a relatively low cost. However, clinical practice suggests that systematic biopsy may be associated with a high false-negative rate (1), and systematic repeat biopsy does not give a satisfactory cancer detection rate (2), in addition to increasing the risk of complications and discomfort to the patient. Additional targeted biopsy in suspicious areas identified by TRUS may improve detection rates, as the sensitivity of conventional TRUS for cancer lesions is relatively low (3,4). Even new imaging techniques such as sonoelastography and contrast-enhanced TRUS do not provide a considerable benefit to the diagnosis of PCa (5,6).

It has been reported that T2-weighted (T2W) magnetic resonance imaging (MRI) and diffusion-weighted imaging (DWI) are useful for diagnosing PCa (7,8). Therefore, there has been an increasing interest in the use of MRI for the diagnosis of PCa. MRI has been used to guide prostatic biopsy successfully, as first reported in 2000; however, MRI guidance is time-consuming and requires specific biopsy equipment (9). It has been hypothesized that TRUS-guided targeted biopsy in suspicious areas identified by MRI has the potential to obtain a high positive rate; however, few patients have been enrolled in studies to investigate this (10,11). The study by Singh *et al* revealed that patient selection, specifically whether they had undergone a negative TRUS-guided biopsy or not and the different interval between two biopsies, influenced the PCa detection rate (12). To study whether MRI is able to increase the PCa detection rate generally, prospective research is required to compare the detection rate between patients undergoing conventional TRUS and those additionally examined by MRI.

The present study was conducted to investigate whether 3-Tesla (3-T) multiparametric MRI prior to biopsy improved the PCa detection rate in patients at their first TRUS-guided biopsies, and to investigate which subgroup had the most evident improvement in PCa detection rate.

## Materials and methods

### Subjects

Between June 2008 and December 2013, 429 consecutive patients (median age, 67 years; range, 45–91 years) underwent 3-T multiparametric MRI prior to their first TRUS-guided prostate biopsies. All patients presented as a result of abnormal digital rectal examination (DRE) findings and/or persistently elevated serum prostate-specific antigen (PSA) levels. Nine cases were excluded due to DWI artifacts resulting from movement of the patient during image acquirement. In the remaining 420 patients, the median PSA level was 9.73 (2.43–35.65) ng/ml and the median prostate volume was 44.82 (21.22–83.22) ml. There were 52 patients with abnormal DRE findings. MRI was performed 2–14 (median 7) days prior to biopsy. The study was approved by the Ethics Committee of Medical College, Shanghai Jiaotong University (Shanghai, China). Signed informed consent was obtained from all patients.

### MRI examination

MRI was performed with a 3-T MRI system (Achieva; Philips, Best, The Netherlands), using a pelvic phased-array coil. First, conventional MRI was performed, including T1-weighted (T1W), T2W and T2W spectral presaturation attenuated inversion recovery (SPAIR). The repetition time/echo time (TR/TE) in T1W, T2W and T2W SPAIR imaging were 353 msec/10 msec, 2,754 msec/80 msec and 2,879 msec/80 msec, respectively. The other main parameters were as follows: thickness, 3 mm; spacing, 1 mm, field of view (FOV), 160×200 mm; matrix, 128×132; number of signal averages (NSA), 3 times. Then, a DWI sequence was performed. The main parameters were as follows: b value, 0 and 1,000 sec/mm^2^; TR/TE 2,500 msec/60 msec; FOV,160×144 mm; matrix, 80×60; thickness, 6 mm; NSA 4 times. An apparent diffusion coefficient (ADC) map was obtained by the computer automatically.

MRI imaging was evaluated by a radiologist who had 10 years’ experience of prostate MR imaging. It was considered abnormal when there were low signal nodules with a mass-like appearance on T2W or T2W SPAIR and high signal nodules on DWI, in either the peripheral zone or the transition zone. The radiologist recorded a confidence level for the probability of malignancy (1, definitely absent; 2, probably absent; 3, undetermined; 4, probably present; 5, definitely present) in different sectors, which was applied as in our previous study (13). Areas of levels 3 to 5 in any MRI imaging were regarded as suspicious. The distances from the suspicious area to the tip, the exterior margin and the posterior border of the prostate were recorded. The prostate volume was calculated using the ellipsoid formula (length × height × width × 0.52) (14). PSA density (PSAD) was calculated by dividing the PSA level by the prostate volume.

### Biopsy protocol

Transperineal prostate biopsy was performed by two operators, guided by transrectal ultrasound. Using a 16-gauge core biopsy gun (Bard Magnum™; Bard Biopsy Systems, Tempe, AZ, USA), a 12-core systematic biopsy (10 cores distributed in a fan-shape from the peripheral zone and 2 cores from the transition zone) was first performed without knowledge of the location of suspicious MRI findings. After two operators had reviewed the information concerning the MRI-suspicious area recorded by the radiologist, targeted biopsy was performed. One or two cores were taken from each suspicious area. Whether the needle was in the correct site was determined by measuring the distance of the needle to the exterior margin and posterior border of prostate, which coincided with the same distances on the corresponding axial MRI.

### Statistical analysis

Differences were analyzed using the Student’s t-test, Kruskal-Wallis test and Chi-square test. P<0.05 was considered to indicate a significant difference. All statistical analyses were performed using SAS software, version 9.13 (SAS Institute, Inc., Cary, NC, USA).

## Results

**Figure 1:**
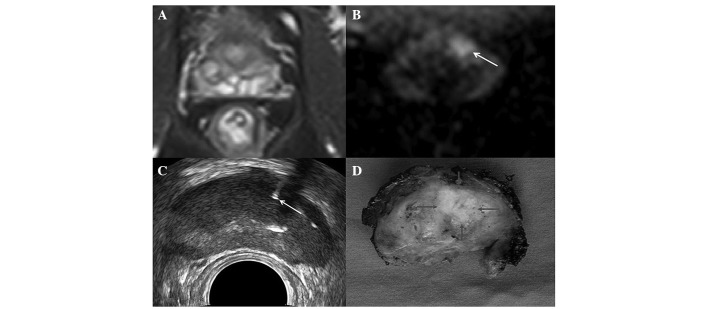
Images obtained from a 68-year-old patient with a PSA level of 8.4 ng/ml. (A) T2W SPAIR showed no suspicious area in the gland. (B) DWI (arrow) showed a hyperintense area in the left transition zone, which was considered suspicious. (C) TRUS-guided prostate biopsy (arrow) in the suspicious area identified by DWI. (D) Radical prostatectomy specimen (arrow) confirmed the presence of PCa. PSA, prostate-specific antigen; T2W SPAIR, T2-weighted spectral presaturation attenuated inversion recovery; DWI, diffusion-weighted imaging; TRUS, transrectal ultrasound; PCa, prostate cancer.

Clinical characteristics of the 420 patients are presented in Table I. PCa was detected in 173 patients (41.2%, 173/420). Among these 173 patients, 41 patients (23.7%, 41/173) were detected by targeted biopsy, but not by systematic biopsy (Fig. 1); 28 patients (16.2%, 28/173) were detected by systematic biopsy, but not by targeted biopsy, and 104 patients (60.1%, 104/173) were detected by both systematic biopsy and targeted biopsy. The increase in the cancer detection rate by targeted biopsy identified by MRI was 9.8% (41/420; P=0.0033). As shown in Table II, among the three groups with PCa detected by different biopsy regimens there were significant differences in serum PSA level, PSAD, prostate volume, DRE findings and TRUS findings, but no differences in age and the biopsy Gleason score.

The efficiency of additional targeted biopsy identified by MRI on the cancer detection rate in different subgroups of patients according to PSA level, PSAD, prostate volume, TRUS findings, and DRE findings is summarized in Table III. The improvement of the cancer detection rate was 9.8% in all cases. There was significant increase in the cancer detection rate in the patient subgroup with a PSA level of 4–10 ng/ml, PSAD of 0.12–0.20 ng/ml^2^, prostate volume >50 ml, negative TRUS findings and negative DRE findings, and the P-values were 0.0256, 0.0133, 0.0099, 0.0027 and 0.0037, respectively.

## Discussion

Since PCa is often multifocal and the volume of prostate sampled by biopsy is relatively small, there is high false-negative rate in conventional TRUS-guided systematic biopsy. Various regimens have been devised to improve the diagnostic yield of prostate biopsies, such as increasing the number of biopsy cores and sampling from the suspicious areas of TRUS for example (15,16). However, the ideal strategy for prostate biopsy has not yet been identified. A study revealed that even saturation biopsy did not significantly improve cancer detection compared with standard biopsy, and was not able to rule out the presence of PCa (17).

MRI has been increasingly used to detect and locate lesions of PCa. 3-T MRI is considered to be superior to 1.5-T MRI with a higher signal to noise ratio and greater spatial resolution (18). Currently, the optimal MRI techniques for PCa are the integrated use of multimodal MRIs, for example, DWI and T2W. DWI has certain advantages, such as fast imaging, without the need for injection of contrast agents. Cancer lesions often show high signal intensity compared with benign tissues on DWI, regardless of whether they are in the peripheral zone or in the transition zone.

Previous studies have found that TRUS-guided repeat biopsies alone result in positive rates of 10–41.1% (1,19–21). When MRI data are added, positive rates for TRUS-guided repeat biopsies of between 24.7 and 40.5% have been observed (10,22,23). It has been suggested that the cancer detection rate might be influenced by previous biopsy techniques and the interval between biopsies (24,25). However, it remains unclear whether additional MRI examination prior to biopsy is useful. Lattouf *et al* observed that MRI prior to TRUS-guided repeat biopsy tended to give higher cancer yields, but the difference was not statistically significant (26). Furthermore, to the best of our knowledge, there are no studies evaluating the advantage of MRI in a large series of cases prior to first biopsy in addition to the standard 12-core systematic biopsy.

In the present study, the overall cancer detection rate was 41.2%, and 41 out of the 173 prostate cancer patients were detected only by targeted biopsy identified by MRI. The improvement of cancer detection rate by targeted biopsy was 9.8% (41/420; P=0.0033). Targeted biopsy identified by MRI may be useful to improve the positive rate on first biopsy. This finding differs from the results of Shigemura *et al* who reported that only 1.04% of cancers had positive cores in MRI targeted biopsy alone (27). This difference may be caused by differences in prostate volume (mean 44.82 vs. 31.9 ml) and serum PSA level (mean 9.73 vs. 8.58 ng/ml) between the two studies. Previous studies have identified patient subgroups with high positive rates in repeat biopsy; there are high false negative rates in patients with a large prostate volume and elevated serum PSA levels (20,28).

In the subgroup analysis of the present study, the improvement of the cancer detection rate by targeted biopsy was 10.1, 12.4, 14.0, 10.9 and 10.1%, respectively, in patients with a PSA level of 4–10 ng/ml, PSAD of 0.12–0.20 ng/ml^2^, prostate volume of >50 ml, negative TRUS findings and negative DRE findings, with P-values of 0.0256, 0.0133, 0.0099, 0.0027 and 0.0037, respectively. A significant increase in the PCa detection rate by targeted biopsy was found in the subgroup with a PSA level of 4–10 ng/ml, while there was no significant difference in the subgroups with a PSA level of <4 ng/ml or >10 ng/ml. This may be explained by the fact that, as a sensitive marker for PCa, a PSA level of <4 ng/ml presents a low incidence of PCa. Despite the addition of MRI data, there is only a small chance of detecting more cancer. By contrast, when the PSA level is >10 ng/ml, the cancer lesions are often so evident that they are detected by systematic biopsy. In patients with a PSA level of 4–10 ng/ml, a considerable number of lesions may remain undetected by systematic biopsy (29), which provides an opportunity for MRI to identify suspicious areas due to its high sensitivity. For similar reasons, the subgroup with a PSAD of 0.12–0.20 ng/ml^2^ obtained the most marked increase in PCa detection rate compared with the other two subgroups. It has previously been reported that a significantly increased prostate volume is one of the important factors responsible for PCa being missed by biopsy (30,31). Since PCa is a multifocal disease, the biopsy technique only provides a limited sample volume of the prostate. The results of the present study showed that the subgroup with a prostate volume of >50 ml obtained the greatest increase in the PCa detection rate. This was in accord with previous studies (30–32). Due to the higher sensitivity of MRI for prostate cancer, it is possible for the detection rate to be improved more significantly in the subgroup of patients with negative TRUS findings or negative DRE findings. For patients with abnormal TRUS or DRE findings, the traditional TRUS-guided biopsy was able to diagnose PCa, and additional targeted biopsies in MRI-suspicious areas were not able to increase the detection rate significantly.

One limitation of the present study was that the accuracy of targeted biopsies may have been reduced since they were not performed with real-time guided-MRI, which could not be used routinely due to it being time-consuming and requiring specific biopsy equipment. A promising imaging technique comprising a fusion of MRI and TRUS may have the ability to improve the accuracy of prostate biopsy (33). Investigation of the value of this technique in our further studies is planned. Another limitation of the present study was that the MRI results were not compared with specimens of radical prostatectomy. Too small a specimen may cause the pathologist to draw a false negative diagnosis. Additionally, the judgment of normal or suspicious MRI imaging was partly operator-dependent. The confidence levels for the probability of malignancy were used to minimize the subjectivity.

In conclusion, this study indicates that MRI may be recommended particularly for the subgroup of patients with a PSA level of 4–10 ng/ml, PSAD of 0.12–0.20 ng/ml^2^, prostate volume of >50 ml, negative TRUS findings and negative DRE findings. 3-T multiparametric MRI has the potential to improve the prostate cancer detection rate on first biopsy.

## Figures and Tables

**Table I:** Characteristics of all patients enrolled in the study.

Characteristics	Prostate cancer	Benign prostate disease	P-value
No. of patients (%)	173 (41.2)	247 (58.8)	
Age (years)	71 (63–77)	65 (58–78)	0.041^a^
PSA level (ng/ml)	11.59 (4.78–19.50)	8.42 (5.34–28.72)	0.017^a^
Prostate volume (ml)	39.57 (25.25–56.01)	48.52 (31.75–70.06)	0.029^a^
PSAD (ng/ml^2^)	0.22 (0.14–0.38)	0.13 (0.07–0.33)	0.010^a^
No. of patients with abnormal DRE (%)	34 (19.7)	18 (7.3)	0.0002
No. of patients with abnormal TRUS (%)	73 (42.2)	45 (18.2)	<0.0001

Data presented are median (interquartile range) or number (%). PSA, prostate-specific antigen; PSA density; DRE, digital rectal examination; TRUS, transrectal ultrasound.

a These values were calculated by t-test; the remaining P-values were calculated by Chi-square test.

**Table II:** Comparison of the characteristics in patients with prostate cancer detected by different biopsy regimens.

Characteristics	TB alone	SB alone	TB + SB	P-value
No. of patients	41 (23.7)	28 (16.2)	104 (60.1)	
Age (years)	68 (61–73)	69 (66–73)	72 (66–79)	0.66^a^
PSA (ng/ml)	7.55 (5.12–10.36)	9.38 (6.21–14.16)	13.78 (3.65–18.17)	0.008^a^
Prostate volume (ml)	47.65 (30.65–62.35)	35.15 (25.06–46.38)	37.57 (24.46–53.93)	0.020^a^
PSAD (ng/ml^2^)	0.13 (0.10–0.17)	0.19 (0.12–0.34)	0.26 (0.15–0.40)	0.028^a^
DRE (No. of patients)				0.028
Normal	37 (90.2)	18 (64.3)	84 (80.8)	
Abnormal	4 (9.8)	10 (35.7)	20 (19.2)	
TRUS (No. of patients)				0.002
Normal	33 (80.5)	12 (42.9)	55 (52.9)	
Abnormal	8 (19.5)	16 (57.1)	49 (47.1)	
Biopsy Gleason score (no. of patients)				0.261
<7	23 (56.1)	15 (53.6)	61 (58.7)	
≥7	18 (43.9)	13 (46.4)	43 (41.3)	

Data presented are median (interquartile range) or number (%). TB, targeted biopsy; SB, systematic biopsy; PSA, prostate-specific antigen; PSA density; DRE, digital rectal examination; TRUS, transrectal ultrasound.

a These values were calculated by *t*-test, and the remaining P values were calculated by chi-square test.

TB, targeted biopsy; SB, systematic biopsy.

**Table III:** Effect of additional targeted biopsy identified by MRI on cancer detection rates.

Characteristics	No. of patients	No. of cancer patients	Increase in the no. of cancer patients	Increase in the positive rate (%)
PSA (ng/ml)				
<4	56	12	3	3/56 (5.4)
4–10	218	84	22	22/218 (10.1)^a^
≥10	146	77	16	16/146 (11.0)
Prostate volume (ml)				
<30	105	52	5	5/105 (4.8)
30–50	172	68	16	16/172 (9.3)
≥50	143	53	20	20/143 (14.0)^b^
PSAD (ng/ml^2^)				
<0.12	80	16	5	5/80 (6.3)
0.12–0.20	185	80	23	23/185 (12.4)^c^
≥0.20	155	77	13	13/155 (8.4)
TRUS				
Normal	302	100	33	33/302 (10.9)^d^
Abnormal	118	73	8	8/118 (6.8)
DRE				
Normal	368	139	37	37/368 (10.1)^e^
Abnormal	52	34	4	4/52 (7.7)
Overall	420	173	41	41/420 (9.8)^f^

These values were calculated by Chi-square test. MRI, magnetic resonance imaging; PSA, prostate-specific antigen; PSA density; DRE, digital rectal examination; TRUS, transrectal ultrasound.

a–e The positive rate was significantly improved, with P-values of 0.0256, 0.0099, 0.0133, 0.0027, 0.0037 and 0.0033, respectively.
